# Direct Blood Dry LAMP: A Rapid, Stable, and Easy Diagnostic Tool for Human African Trypanosomiasis

**DOI:** 10.1371/journal.pntd.0003578

**Published:** 2015-03-13

**Authors:** Kyoko Hayashida, Kiichi Kajino, Lottie Hachaambwa, Boniface Namangala, Chihiro Sugimoto

**Affiliations:** 1 Division of Collaboration and Education, Research Center for Zoonosis Control, Hokkaido University, Sapporo, Japan; 2 National Research Center for Protozoan Diseases, Obihiro University of Agriculture and Veterinary Medicine, Obihiro, Japan; 3 Department of Internal Medicine, University Teaching Hospital, Lusaka, Zambia; 4 Department of Paraclinical Studies, School of Veterinary Medicine, University of Zambia, Lusaka, Zambia; United States Food and Drug Administration, UNITED STATES

## Abstract

Loop-mediated isothermal amplification (LAMP) is a rapid and sensitive tool used for the diagnosis of a variety of infectious diseases. One of the advantages of this method over the polymerase chain reaction is that DNA amplification occurs at a constant temperature, usually between 60–65°C; therefore, expensive devices are unnecessary for this step. However, LAMP still requires complicated sample preparation steps and a well-equipped laboratory to produce reliable and reproducible results, which limits its use in resource-poor laboratories in most developing countries. In this study, we made several substantial modifications to the technique to carry out on-site diagnosis of Human African Trypanosomiasis (HAT) in remote areas using LAMP. The first essential improvement was that LAMP reagents were dried and stabilized in a single tube by incorporating trehalose as a cryoprotectant to prolong shelf life at ambient temperature. The second technical improvement was achieved by simplifying the sample preparation step so that DNA or RNA could be amplified directly from detergent-lysed blood samples. With these modifications, diagnosis of HAT in local clinics or villages in endemic areas becomes a reality, which could greatly impact on the application of diagnosis not only for HAT but also for other tropical diseases.

## Author Summary

Human African trypanosomiasis (HAT) is an endemic protozoan disease affecting many African countries, predominantly in rural areas where the tsetse fly vector is present. HAT diagnosis currently relies on conventional microscopy techniques, which provide relatively low sensitivity and are often unable to distinguish between the two different HAT-causing subspecies, *T*. *b*. *rhodesiense* and *T*. *b*. *gambiense*. A delay in HAT diagnosis can result in disease progression from an early stage (hemolymphatic) to a late stage (meningoencephalitic); therefore, a rapid and sensitive diagnostic test is crucial to its successful treatment. In this study, we developed a simple and highly sensitive molecular technique for HAT diagnosis. Loop-mediated isothermal amplification (LAMP) is a method that rapidly amplifies DNA with high sensitivity under isothermal conditions. We optimized and modified the technique by stabilizing the reagents and simplifying the sample preparation for the diagnosis of HAT. The whole protocol is so simple that local staff can perform it without the need for costly equipment and expertise.

## Introduction

Human African trypanosomiasis (HAT), also known as “sleeping sickness”, is caused by the protozoan parasites *Trypanosoma brucei rhodesiense* or *Trypanosoma brucei gambiense*. Majority of reported HAT cases are caused by *T*. *b*. *gambiense* in Western and Central Africa, and sporadic HAT cases in East Africa are caused by *T*. *b*. *rhodesiense*. Infection with *T*. *b*. *gambiense* is characterized by chronic disease progression, whereas *T*. *b*. *rhodesiense* causes a more acute infection [1]. In both subspecies, parasites circulating in the blood or lymphatic system often cause general flu-like clinical signs, including fever, general malaise, anemia, lymphadenopathy, and arthralgia, and may therefore be misdiagnosed as other febrile illnesses, such as malaria. In the initial stage, HAT is curable with drugs, such as pentamidine and suramin [2]. In the second stage, trypanosomes eventually cross the blood-brain barrier to invade the central nervous system (CNS), leading to the typical symptoms of sleeping sickness, such as sleep cycle disturbances. In the final stage, patients deteriorate into a coma and the disease is fatal if left untreated. Melarsoprol, and more recently, NECT (nifurtimox–eflornithine combination therapy) for *T*. *b*. *gambiense* [3], is the drug of choice for the second stage of the disease; however, melarsoprol can be associated with severe side-effects and even death [2], and NECT is unfortunately less effective to *T*. *b rhodesiense*. Therefore, early HAT detection is crucial for the effective treatment and management of this disease.

The loop-mediated isothermal amplification (LAMP) [4], [5], will be useful methods for rapid and sensitive HAT diagnosis in under-equipped laboratory. LAMP can amplify parasite DNA from blood or spinal fluids with very low parasitemia within 30 minutes under isothermal conditions [6], [7]. The analytical sensitivity of the LAMP assay targeting a repetitive insertion mobile element (RIME) is equivalent to 0.01 trypanosomes/ml compared to 0.1 to 1000 trypanosomes/ml for classical PCR [6]. Based on this technique, a commercial LAMP reagent kit, Loopamp Trypanosoma brucei assay (Eiken Chemical Co LTD, Japan, in collaboration with the Foundation for Innovative New Diagnostics [FIND], Geneva, Switzerland) has been developed [8], [9]. This kit comes in a ready-to-use format, with dried reagents and positive/negative controls. One of the major obstacles for the application of LAMP in distant surveillance sites, especially in the tropics, has been the difficulty in maintaining the cold chain; therefore, the concept of dried reagents is innovative. This LAMP kit is rapid and highly sensitive, however, it is expensive, still relies on a well-equipped laboratory for the successful extraction of DNA from blood samples and the methodology for making dried reagents is absolutely confidential. To solve these issues, we refined LAMP system for HAT diagnosis that is cost effective and stable at high temperature. In our system, lysed blood can be used directly with high detection sensitivity. This dry LAMP system will be widely applicable in the field or for bedside diagnosis even in areas lacking adequate infrastructure.

## Materials and Methods

### *Trypanosoma brucei rhodesiense* strains and DNA

The *T*. *brucei rhodesiense* strain UTH2012 from Zambia [10] was used in this study. It is an isolate from human patient peripheral blood. The blood was injected and propagated in NIH Swiss mice (n = 3), and the bloodstream form of the parasite was purified using a DE52 anion-exchange column [11]. The *T*. *b*. *rhodesiense* isolates were adapted to and maintained in HMI-9 medium supplemented with 20% fetal bovine serum [12]. The DNA was prepared using the FujiFilm QuickGene DNA Whole Blood Kit (FujiFilm, Tokyo, Japan) according to the manufacturer’s instructions. The extracted DNA was quantified using the Quanti-iT ds DNA HS assay in a Qubit fluorometer (Invitrogen, Carlsbad, CA) and stored at −20°C.

### Ethical clearance

The use of mice were approved by the Biomedical Research Ethics Committee of University of Zambia under the JICA-JST SATREPS project “Establishment of Rapid Diagnostic Tools for Tuberculosis and Trypanosomiasis and Screening of Candidate Compounds for Trypanosomiasis” and the experiments were carried out according to the guidelines for proper conduct of animal experiments by the Science Council of Japan (2006).

### LAMP primers

**Fig 1:**
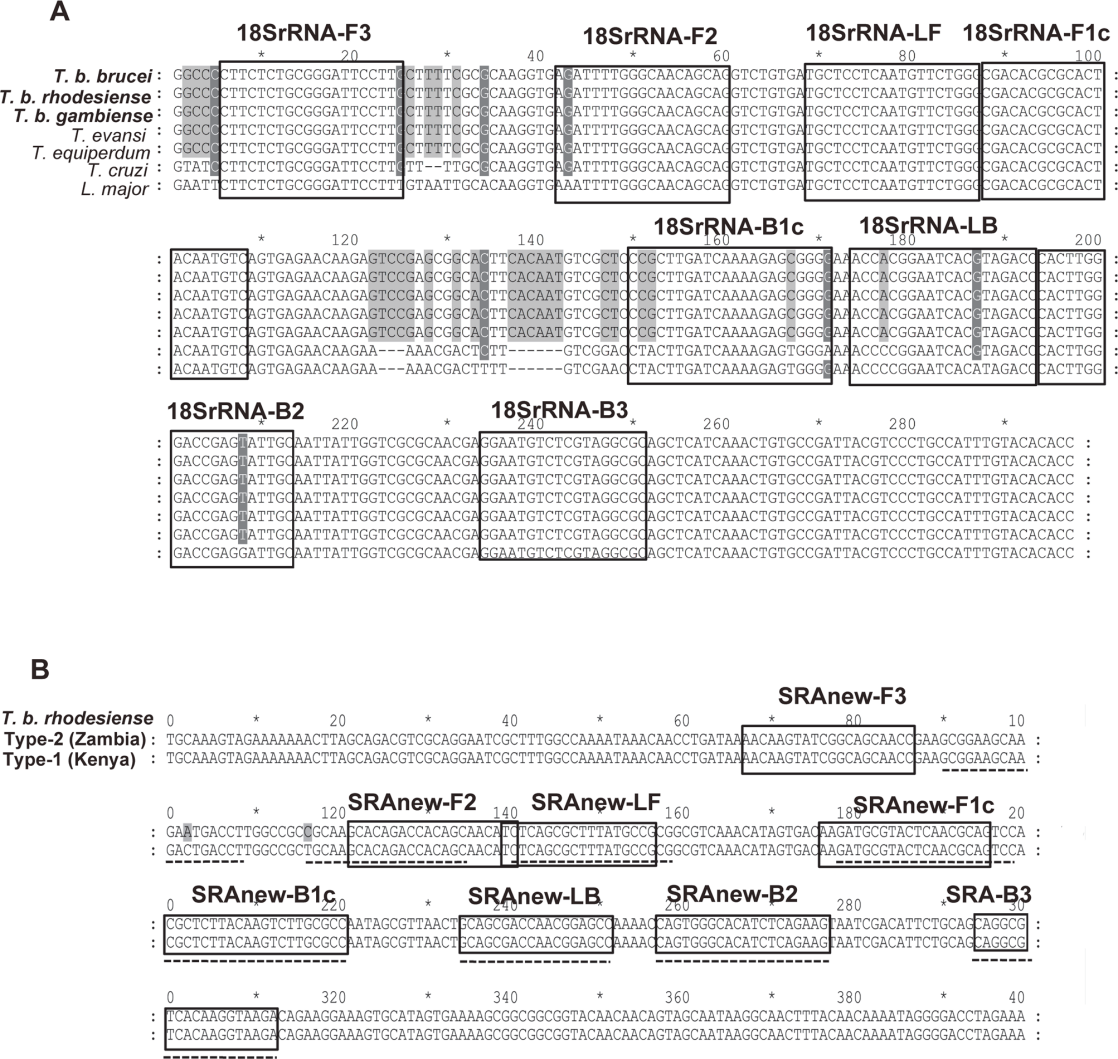
LAMP primers design for *T*. *b*. *rhodesiense* nucleotide detection. (A) Nucleotide sequence alignments of *18S-rRNA* genes of the seven Trypanosoma species. Representative*18S-rRNA* sequences in each species are aligned with clustalW. *T*. *brucei brucei* strain 927 (AL929603), *T*. *brucei rhodesiense* isolate UTRO 2509 (AJ009142), *T*. *brucei gambiense* DAL972 (FW554966), *T*. *evansi* isolate E110 (AJ009154), *T*. *equiperdum* isolate STIB 818 (AJ009153), *T*. *cruzi* marinkellei culture collection TCC/USP (FJ001665) and *Leishmania major* strain Friedlin (FR796423). Primer recognition sites are indicated with primer names. Gray boxes indicate non-conserved nucleotides. (B) Nucleotide sequence alignments of the target region in the two types of *SRA* genes. SRA type 1 was isolated in Kenya (AJ345057) and SRA type 2 was isolated in Zambia (AJ345058) [13,14]. Because previously reported primers [7] had polymorphisms in SRA type 2 (shown by the dotted line), a conserved region was selected as the new primer recognition site.

Three LAMP primer sets (Table 1) were used to detect *T*. *b*. *rhodesiense*. The LAMP primer sets were designed as previously described by Notomi et al. [4]. The multicopy repetitive insertion mobile element (RIME) primers specific to the *Trypanosoma brucei* s.l. subspecies were adapted from a previous report [6] with slight modifications in some of the them. The primers targeting 18S-rRNA for *T*. *brucei* s.l. subspecies were newly designed in this study for reverse-transcription (RT)-LAMP. The sequences of the 18S-rRNA targeting region are also conserved in *T*. *evansi*, and *T*. *equiperdum* (Fig. 1). The human serum resistance-associated gene (SRA) primers specific to *T*. *b*. *rhodesiense* were also modified in this study so that both currently known forms of SRA, Type-1 found in Uganda, Kenya, Tanzania, and Type-2 found in Zambia, Ethiopia, and Tanzania [13], [14] could be amplified (Fig. 1). These primer sets were evaluated by real-time LAMP and also melting curve analysis using the Rotor-Gene 3000 thermal cycler (Corbett Research, Sydney, Australia) to monitor the reaction, and optimal amplification time and temperature for primer sets were determined.

**Table 1:** LAMP primer sequences designed for *T. brucei* subspecies (18S-rRNA and RIME) or *T. b.* *rhodesiense* (SRA) used in this study.

18S-rRNA	TgrRNA18S-FIP	GACATTGTAGTGCGCGTGTCGAGATTTTGGGCAACAGCAG
	TgrRNA18S-BIP	CCGCTTGATCAAAAGAGCGGGGGCAATACTCGGTCCCAAGTG
	TgrRNA18S-LF	CCCAGAACATTGAGGAGCA
	TgrRNA18S-LB	ACCACGGAATCACGTAGACC
	TgrRNA18S-F3	CTTCTCTGCGGGATTCCTTG
	TgrRNA18S-B3	GCGCCTACGAGACATTCC
RIME	RIME-FIP	GGAATACAGCAGATGGGGCGAGGCCAATTGGCATCTTTGGGA
	RIME-BIP	AAGGGAGACTCTGCCACAGTCGTCAGCCATCACCGTAGAGC
	RIME-LF	GCCTCCCACCCTGGACTC
	RIME-LBm	CCAGACCGATAGCATCTCAG
	RIME-F3	CTGTCCGGTGATGTGGAAC
	RIME-B3	CGTGCCTTCGTGAGAGTTTC
SRA	SRA-FIPm	CTGCGTTGAGTACGCATCTTGCACAGACCACAGCAACATC
	SRA-BIP	CGCTCTTACAAGTCTTGCGCCCTTCTGAGATGTGCCCACTG
	SRA-LFm	CGGCATAAAGCGCTGAGA
	SRA-LB	GCAGCGACCAACGGAGCC
	SRA-F3m	AACAAGTATCGGCAGCAACC
	SRA-B3	TCTTACCTTGTGACGCCTG

### Colori-Fluorometric Indicator (CFI)

As an indicator for the LAMP reaction, we developed a combination of dyes that we have called a “colori-fluorometric indicator (CFI)”. The CFI stock solution consists of 3 mM hydroxyl-naphtol blue (HNB; MP Biomedicals, Aurora, OH) and 0.35% v/v GelGreen (10,000× Sol, Biotium, Hayward, CA) dissolved in distilled water.

### Drying procedure of LAMP reagents

All LAMP reagents were placed in a single 0.2 ml microtube and air-dried. The procedure was as follows.

#### Step 1) Primer/CFI dry-up step

**Fig 2:**
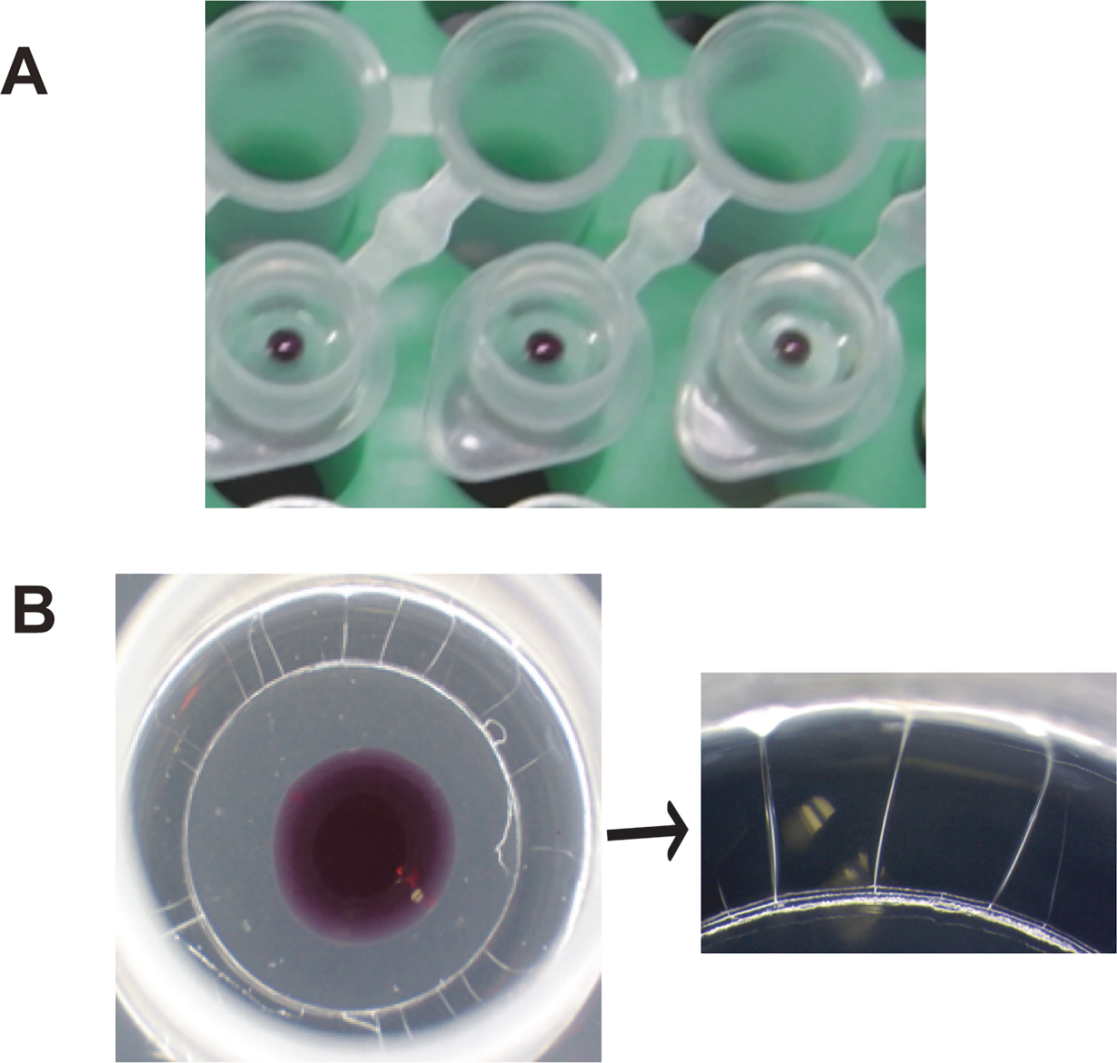
The appearance of vitrified reagent of Dry HAT LAMP. (A) The drying process of Dry LAMP. The enzyme solution and primer/indicator mix were put on the inner side of the cap separately. (B) Close-up picture of dried LAMP solutions. The vitrified enzyme solution displayed several cracks.

100 μM stocks of six primers (FIP, BIP, F3, B3, LF, and LB) were prepared in DDW. Primer mixtures consisting of 0.4 μl each of FIP and BIP, 0.05 μl each of F3 and B3, and 0.2 μl each of LF and LB, 0.56 μl of 2 M trehalose (Wako, Japan), and 0.14 μl of 50% Glycerol (Nacarai tesque, Japan) were prepared. Two μl of primer mixture and 1 μl of the CFI stock solution were mixed in each tube and placed in the center of the lid of a 0.2 ml tube (Fig. 2). The final primer concentration of each primer in a reaction is 1.6 μM for FIP and BIP, 0.2 μM for F3 and B3, and 0.8 μM for LF and LB. The mixture was dried for approximately 30 min under a flow of clean air.

#### Step 2a) *Bst* polymerase/dNTPs dry-up step

After step 1 was completed, 1.5 μl of 2M trehalose (Wako), 1.4 μl of dNTPs (25 mM each; Nippon gene, Japan), 0.05 μl of *Bst*2.0 WS DNA polymerase (120 U/μl; NEB Japan, Japan), and 0.3 μl of *Bst*2.0 WS DNA polymerase (8 U/μl; NEB Japan) were mixed and placed in the peripheral part of the tube lid (Fig. 2). The mixture was placed under a flow of clean air for 15 min.

#### Step 2b) *Bst* Polymerase/dNTPs/ RT-polymerase dry-up step

For the RT-LAMP reaction, this step was carried out instead of step 2a, after step 1 was completed. 1.6 μl of 2M trehalose, 1.4 μl of dNTPs (25mM each), 0.05 μl of *Bst* 2.0 WS DNA polymerase (120 U/μl), 0.25 μl of *Bst* 2.0WS DNA polymerase (8 U/μl), 0.1 μl of RNase inhibitor (40 U/μl; Nakaraitesque), and 0.04 μl of AMV reverse transcriptase (20 U/μl; Nippongene) were mixed and air-dried for 15 min under a flow of clean air.

#### Step 3) Final dry-up step

The tubes were further dried in a container with phosphorus oxide (P_2_O_5_) and silica gel for >24 h under a vacuum until they were completely dry. After this step, the tubes were stored in an aluminum bag with zeolite molecular sieves.

### LAMP reaction

The dried LAMP reagents were used as follows.

- **Purified DNA.** When purified genomic DNA from the culture or blood was used as a reaction template, the DNA plus 1 μl of 25 × LAMP buffer (500 mM Tris–HCl [pH 8.8], 250 mM KCl, and 100 mM MgSO_4_), 1.5 μl of 100mM MgSO_4_ (final conc. 7mM), and 0.1% TritonX-100 in DDW were added to make a 25-μl reaction mix. The tubes were turned upside down, and mixed well so that the dried reagents were completely reconstituted.
- **Blood-direct method.** We optimized the simple human blood lysis procedure for the LAMP reaction to simplify the DNA/RNA preparation process. A total of 200 μl of human blood in a heparin tube were transferred into a tube containing 1,800 μl of blood lysis buffer (0.1% TritonX-100 in DDW) and mixed well. Then, 10 μl of lysed blood were used for the reaction within 5 minutes. As described above, 1 μl of 25 × LAMP buffer, 1.5 μl of 100 mM MgSO_4_ (final conc. 7 mM), and 0.1% TritonX-100 in DDW were added to 25-μl reaction tubes. When the blood was collected in an EDTA tube, the magnesium concentration of the LAMP reaction buffer was adjusted to a final concentration of 7.8 mM (1.7 μl of MgSO4 were added). After the reaction mixture was prepared, the tubes were incubated at 60°C for 45 min. As a negative control, extracted DNA sample was replaced by DDW, or trypanosoma free human blood lysate was used for direct blood assay.

### Fluorescence detection

DNA products amplified using LAMP were visualized using GelGreen contained in the CFI dye. Since the excitation/emission maxima of GelGreen are 500 nm/530 nm, respectively, the reactions were visualized using either a blue LED illuminator with a 470 nm light, PrepOne Sapphire 2 (Alliance Biosystems, Japan), or by using a Rotor-Gene 3000 thermocycler to monitor the reaction in real time using the FAM/green channel. We also developed a battery-driven hand-made LED illuminator that emits 500 nm wavelength light, which was suitable for field or bedside use. The design of the hand-made LED illuminator is shown (S1 Fig.).

## Results

### Development of a dual indicator, CFI

GelGreen is a fluorescent DNA intercalator with decreased carcinogenicity, originally developed for in-gel staining of nucleic acids. Because it shows inhibitory effects on most DNA polymerases commonly used in PCR reactions, it is usually used post-PCR staining. However, we found it did not affect the activity of *Bst* DNA polymerase at the concentration normally used in in-gel staining even if incorporated into the LAMP reaction mixture (S2 Fig.). Thus, we used this reagent in combination with HNB for the LAMP assay and named it colori-fluorometric indicator (CFI). Using CFI, the LAMP reactions could be detected in two ways: either by detecting amplified DNA products with GelGreen or by a decreased Mg2+ concentration with HNB. The resulting fluorescence was visible under a blue-green (λ = 500 nm) LED illuminator as described in the Materials and Methods. The color of HNB changed from violet to blue during the progression of the LAMP reaction as the Mg^2+^ ion concentration decreased by forming insoluble magnesium pyrophosphate. These color changes were easily visible by the naked eye. The sensitivity, as determined by a ten-fold detection limit was comparable to that of calcein, a commonly used color-development reagent for LAMP (S2 Fig.).

### Preparation of dried reagents with trehalose/glycerol

LAMP reagents were dried by adding 3 μmol of trehalose, a disaccharide widely known for its efficacy in preserving proteins in a dry state at room temperature. As commercially available *Bst* DNA polymerase already contains a certain concentration of glycerol, the final mixture contained two components as protectants. The amount of glycerol was optimized at 22.1% (w/v) by mixing two different concentrations of *Bst* DNA polymerases; *Bst* 2.0 WarmStart DNA Polymerase 8,000 U/ml (NEB M0538L) and 120,000 U/ml (NEB M0538M), so that proper drying time and enzyme stability were achieved. The primer/CFI solution and enzyme mixture were dried separately onto the lid of a single tube (Figs. 2A and 1B), so the reaction started just after all the components (primer/enzyme/template) were mixed and incubated at the required temperature. In this formulation, called “Dry LAMP”, the liquid solution changed to a glass phase form after drying and was retained at up to 55°C (Fig. 2B).

### Sensitivity of Dry HAT-LAMP assay

**Fig 3:**
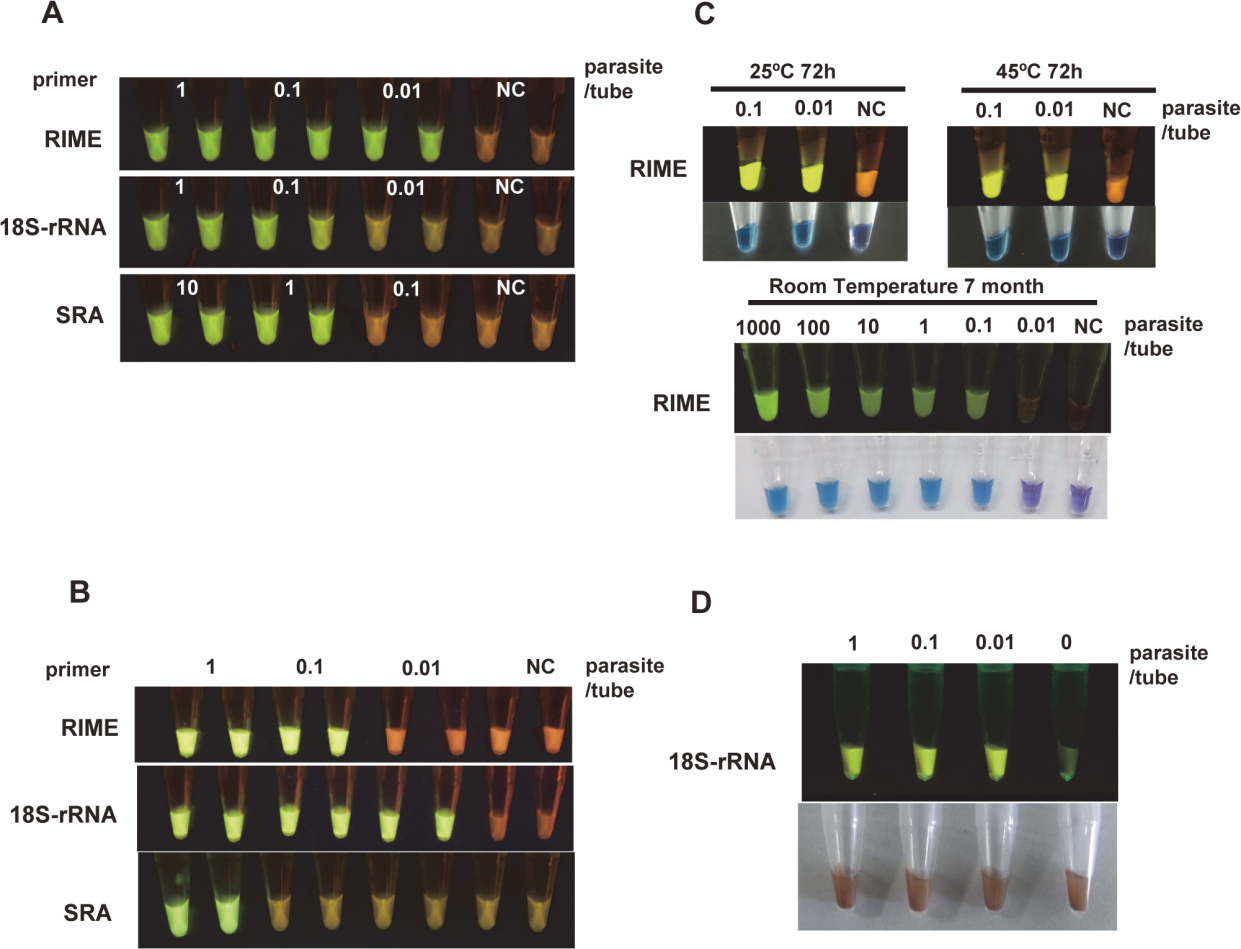
Sensitivity and thermostability of HAT-LAMP. (A) Sensitivity comparison of RIME, 18S-rRNA, and SRA primer in LAMP using extracted and purified parasite DNA. The RIME primer set detected 0.01 parasites/tube. (B) Sensitivity comparison of primer sets in RT-LAMP. The 18S-rRNA primer set detected ten live parasites per ml of TBS, equivalent to 0.01 parasites/tube. (C) Thermostability tests of Dry LAMP using extracted and purified parasite DNA. The sensitivity did not change after a 72-h heating load (upper) or 7-month storage (lower). (D) Sensitivity of the 18S-rRNA primer set in RT-LAMP remained unchanged in the presence of 1 μl of human blood. The reaction can be detected by fluorescence. 1 parasite DNA = 0.1 pg DNA.

Using ten-fold serially diluted *T*. *b*. *rhodesiense* genomic DNA as a template, the sensitivity of three different HAT-LAMP systems was determined. The detection limits of the RIME, 18S-rRNA, and SRA-LAMP were 0.01, 0.1, and 1 parasite equivalent DNA per reaction, respectively (Fig. 3A), with the contents of DNA in a single parasite estimated at 0.1 pg DNA [15].

Theoretically, a higher sensitivity was expected if a highly transcribed gene was selected as a target. Therefore we tried to target transcripts from ruptured parasites. For this purpose, reverse transcriptase (0.625 U) and RNase inhibitors were added to each reaction tube. The sensitivity of RT-LAMP for each primer set was measured as listed below. For RNA detection, serially diluted live *T*. *b*. *rhodesiense* parasites suspended in PBS were used as the starting material. The parasite suspension was lysed with 0.1% TritonX-100 and the lysate was immediately transferred to an assay tube. The detection sensitivity limits of RIME-, 18S-rRNA-,and SRA-LAMPs using the dried RT-LAMP system were, 0.1, 0.01 and 1 parasite per tube, respectively (Fig. 3B).

### Cross reactivity of HAT-LAMP assay

We assessed cross reactivity of dry HAT-LAMP system with three primer sets originally developed for *T*. *b*. *rhodesiense* to human infective other related parasites, namely *Trypanosoma brucei gambience* (IL2343), two *Trypanosoma cruzi strains* (Y and Tulahuen strain), *Leishmania donovani* (MHOM/SU/62/2S-25M-C2), and *Plasmodium falciparum* (3D7). As shown in S3 Fig., RIME-LAMP reacted with *T*. *b*. *gambiense*, and 18S rRNA targeting primer sets showed positive reactions for all *Trypanosoma* species (*T*. *b*. *gambiense*, *T*. *cruzi* Y and Tulahuen) as also evidenced by the bioinformatics analysis (Fig. 1). SRA primer sets only reacted with *T*. *b*. *rhodesiense*, indicating possible molecular technique to differentiate two forms of HAT subspecies. No cross-reactivity was observed to *L*. *donovani*, *P*. *falciparum* or human DNA (S3 Fig.).

### Stability of Dry LAMP

To estimate the thermostability of Dry LAMP, the tubes for *T*. *b*. *rhodesiense* RIME-LAMP detection were incubated on a heat block adjusted at 25°C or 45°C for 72 hours. The sensitivities of heated and non-heated Dry LAMP were comparable (Fig. 3C). Long-term storage stability was also tested. Dry LAMP test tubes stored with a desiccant in a light-resistant bag at an ambient temperature between 20°C and 30°C for 7 months decreased sensitivity to *T*. *b*. *rhodesiense* RIME, but this was still high enough to detect the equivalent of 0.1 parasite DNA per reaction (Fig. 3C).

### Simplification of sample preparation for Dry LAMP

As indicated in previous reports [6], RIME primer sets showed the best sensitivity when extracted parasite DNA was used as a template. However, the DNA extraction process was often associated with problems due to the need for DNA extraction kits and equipment such as a centrifuge, which are not always available at local clinics or diagnostic laboratories in developing countries. Hemoglobin contained in erythrocytes usually acts as an inhibitor of PCR enzymes. Fortunately, *Bst* DNA polymerase is known to be relatively resistant to hemoglobin contamination [7], [16]. Therefore, we decided to pursue the possibility of making the DNA/RNA preparation process as simple as possible. To mimic infected blood, we prepared samples by mixing heparinized human blood with serially diluted parasite suspensions, diluted ten times with a lysis buffer containing Triton X-100. Ten μl of the lysate were transferred into a RT-LAMP test tube prepared with the 18S-rRNA-targeting primer set (Fig. 3D). As indicated in Fig. 3, detection sensitivity of 18S-rRNA primer set for RT-LAMP (Fig. 3B) was equivalent to that obtained when RIME primer set and purified DNA were used (Fig. 3A).

## Discussion

Currently available diagnostic methods for HAT are based on the microscopic visualization of parasites in the blood, either by Giemsa-staining thin or thick smears or by micro-capillary centrifugation. There are two major problems with these methods: one is low sensitivity and the other is the need for a microscope and an experienced technician. Unless the parasites are concentrated, the sensitivity using microscopy on wet-smears is less than several thousand parasites per ml of blood [17], which often cause fatal delays in treatment. As such, capillary tube centrifugation (CTC, WOO) [18], quantitative buffy coat [19], and mini anion exchange centrifugation technique (mAECT) [20], and the modified single centrifugation method (MSC) [21] have been established. These parasite concentration techniques are simple and straightforward, with high sensitivity detecting <2 trypanosomes per ml of CSF in the case of MSC, but still rely on expensive devices like centrifuge and microscope with electricity, and differentiation between *T*. *b*. *gambiense* and *T*. *b*. *rhodesiense* is not possible.

LAMP is an ideal molecular diagnostics method, especially in under-equipped conditions, because it does not require costly or specialized devices such as thermal-cyclers. Most of the clinical demands for this simple technique exist in developing countries. Its rapid and highly sensitive diagnostic capacity should be applied to the early diagnosis of HAT patients. Until now, many reports concerning HAT-LAMP diagnosis have been published [6], [7], [9], [10], but most of these studies were performed in well-equipped laboratories and never applied to field or bedside studies, particularly in HAT-endemic countries. In this study, LAMP methods were modified for more practical uses. Our major improvements were: i) the development of stable and cost-effective reaction indicators; ii) overcoming transport issues by vitrification of heat-labile compounds and enzymes; iii) the simplification of the sample preparation step; iv) the optimization of primer sequences to increase sensitivity and specificity; and v) designing a battery-driven portable LAMP reaction detector.

Firstly, we developed a dual indicator of the LAMP reaction, named CFI. A conventional color-development reagent, calcein, which has been optimized to minimize background noise, has been marketed under the brand Fluorescent Detection Reagent (FD) by Eiken. It can be premixed into a reaction with no inhibitory effects. In theory, it does not detect amplified DNA products but indirectly detects concomitant reactions such as Mg2^+^ ion depletion in the reaction tube. Because of this, calcein is not suitable for use in EDTA-containing blood directly. To avoid chelate contamination in the specimens, a heparin anticoagulant or DNA purification step are recommended. On the other hand, GelGreen contained in CFI directly detects amplified DNA; therefore LAMP is possible even in samples containing EDTA or citric acid as anticoagulants. The sensitivity of CFI is almost equal to that of FD. Other DNA intercalators, such as SYTO-9, are available for real-time LAMP [7], but are very expensive. SYBR Green can also be used [22], but it must be added after the reaction as it reduces the sensitivity of LAMP. By contrast, GelGreen is very cost-effective compared to the other two DNA-binding reagents and even to FD. Therefore the Dry LAMP reagents used in this study costs up to one dollar per tube, including enzymes, buffers, RNase inhibitor and CFI. The use of HNB as a LAMP reaction indicator has already been reported by Goto *et al*., [23]. Along with its detectability under visible light, HNB can reduce background signals when GelGreen is used under blue-green emission light. In particular under blue-green light, tubes without the LAMP reaction glow red because emission from HNB is dominant, while tubes with amplified DNA glow green because HNB emission is replaced by bright yellow-green fluorescence from GelGreen+dsDNA. The best advantage of CFI is its applicability in blood containing the LAMP reaction. Using CFI, the lysed blood can proceed to LAMP assay directly without any purification step. The fluorescent differences between a positive and a negative sample of direct blood are quite obvious. In a negative tube, HNB completely eliminates background fluorescence from GelGreen.

Secondly, we applied vitrification technology of enzymes using trehalose. Trehalose is a disaccharide used as a protein and phospholipid stabilizer during drying. Mixtures containing a very high concentration of trehalose change their phase from liquid to amorphous solids, which hold trapped biological molecules retaining native structures during desiccation [24]. We have optimized the drying conditions of LAMP reagents, and finally managed to dry them in a single reaction tube without reducing sensitivity. Thermostability and shelf life under ambient temperature have also been verified. This technology enables us to transport LAMP kits to areas where the cold chain is not easily available.

**Fig 4:**
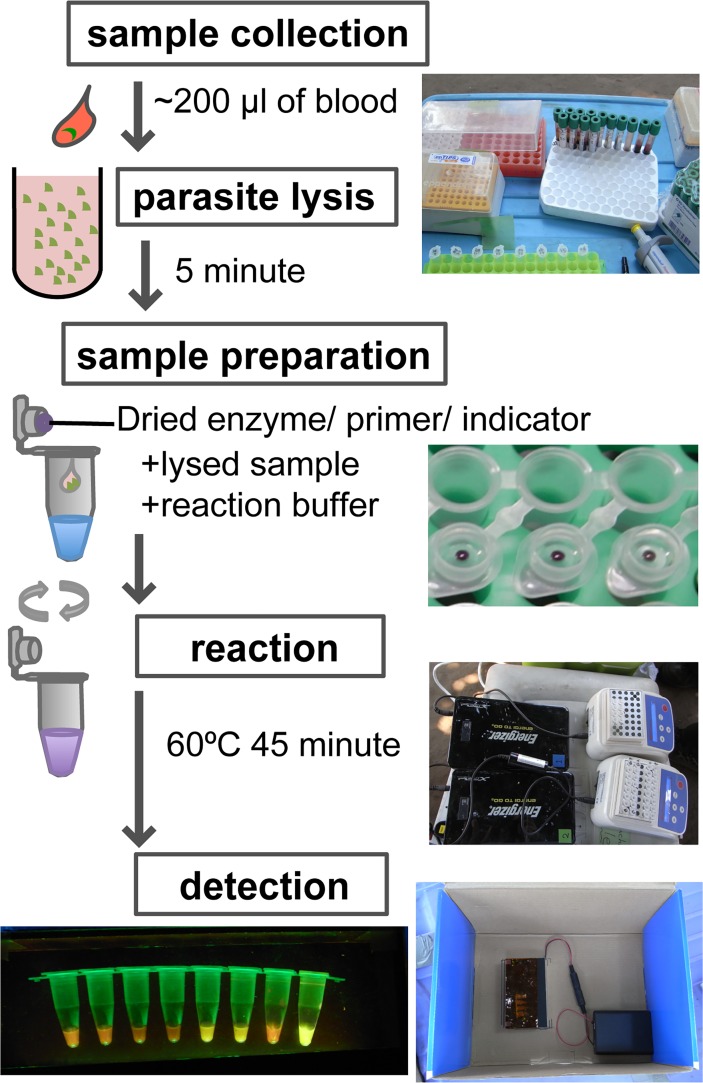
A series of operations of 18S-rRNA RT-LAMP screening test. Two hundred μl of blood were diluted in 1,800 μl of lysis buffer (10× dilution), then 10 μl of lysed blood were transferred into a Dry LAMP tube with 15 μl of reaction buffer within 5 minutes. After a 45-min incubation at 60°C using a portable battery-supplied heat block, the LAMP reaction was evaluated by a portable reaction detector.

As a simplified sample preparation for LAMP, boiling methods have been proposed [7], [16]. These use clear aqueous components containing DNA obtained by centrifugation after thermal aggregation to precipitate hemoglobin in blood samples. Compared to the use of DNA extraction kits, boiling methods can reduce cost, time, and effort required for the preparation of clinical samples; however, they still require basic equipment such as a centrifuge and a heat block over 90°C. Our solubilization method with Triton X-100 is easier, does not require any electric devices, and allows us to carry out LAMP at the bedside or sampling site (Fig. 4). This simple blood preparation procedure was not applicable for conventional color-development reagent calcein (FD).

Selection of the parasite target gene and optimization of the LAMP primer sets are other key elements for a sensitive and specific diagnosis. As approximately 500 copies of RIME sequences are distributed across the *T*. *b*. *rhodesiense* genome, LAMP primer set targeting this gene is considered to have the highest sensitivity. However, DNA extraction and shearing the large genomic DNA are necessary to achieve the best results from the RIME-LAMP reaction. Because RNA containing 18S-rRNA is abundantly present in cells and easily released by lysis, it could be another promising target. In this report, we added a reverse transcriptase into LAMP for the sensitive detection of parasites in the blood. In 4 independent experiments, 18S RT-LAMP detected *T*. *b*. *rhodesiense* RNA 8/8 (100%) in 1 parasite/tube, 8/8 (100%) in 0.1 parasites/tube and 7/8 (87.5%) in 0.01 parasites/tube respectively, that is RT-LAMP using the 18S-rRNA primer set detects as few as one parasites per 100 μl of blood. Thus, RIME primer sets and 18S rRNA primer set showed best performance (0.01 parasite per reaction tube) using purified DNA and blood lysis method, respectively. SRA primer set showed less sensitivity detecting 1 parasite per reaction tube both in purified DNA and lysis method, but will be useful for differentiation of two forms of HAT, *T*. *b*. *gambiense* and *T*. *b*. *rhodesiense*. Blood instillation method into FTA cards have been commonly used in LAMP assays [16], [25]. However, there is the possibility of missing parasite-containing areas, especially in sampling from the FTA-dried blood with a low parasitemia, such as in the case of chronic *T*. *b*. *gambiense* infection [17]. If DNA is prepared from a larger volume of blood with a low parasitemia, the possibility of missing parasites decreases. In this context, lysis of whole blood with 10 volumes of Triton X solution may increase the sensitivity of the technique.

It is already known that there are two subtypes of SRA: type 1 (Accession no: AJ345057) found in Uganda, Kenya, and Tanzania; and type 2 (Accession no: AJ345058) found in Zambia, Ethiopia, and Tanzania [13], [14]. As the existing primer set for SRA was designed for type 1, we have re-designed new SRA primer sets suitable for detecting both type 1 and type 2 *T*. *b*. *rhodesiense*. This has increased the sensitivity ten-fold compared to the previous primer sets (S4 Fig.).

Primer-dimer or primer-multimer formation is one of the causes of non-specific reactions in LAMP. To avoid this, we separated the primers from the enzyme and adopted WarmStart *Bst* polymerase. We also optimized the sequences of all primer sets with the use of a Rotor-Gene 3000 thermal cycler by screening a number of primer combinations and selected a most rapid, sensitive, and specific primer set.

Especially for the direct blood application, we assembled a battery-driven portable LAMP reaction detector. In the case of blood samples used for LAMP diagnosis, color detection of HNB by the naked eye is hindered, while fluorescence detection is not affected. Therefore, we designed a portable transilluminator powered by AA batteries or a car 12V port and assembled it using acrylic plates and LEDs. This was suitable for both field and bedside use. A blue-green light emitted from the LED also improved the safety of LAMP detection because the light was less harmful to the eyes than UV or blue around 440nm wavelength light, along with the use of harmless dyes.

In this study, we refined LAMP detection system, making the feasibility of LAMP for bedside diagnosis and field surveillance. Our LAMP system can be applied to a wide range of other infectious diseases and therefore paves the way for possible utilization of rapid molecular diagnostic tests at point of care stations in resource poor countries.

## Supporting Information

S1 Fig (A) Light emitting diodes (LEDs) with a 505 nm dominant wavelength used in the LAMP reaction detector (RD). (B) Diagrammatic illustration of the portable RD. Emission light was coalesced into 500 nm wavelength by a gelatin film band path filter (FUJIFILM BPB-50). (C) Overall picture of LAMP RD. (D) Views of sample tubes in the RD. Naked eye (upper), emitted in the lighted (middle) or in the dark (lower) environment. The third tube from the left is reaction positive and the others are all negative.
(TIF)

S2 Fig Comparison of detection sensitivity among LAMP reaction indicators. (A) GelGreen; (B) Hydroxy-naphtol blue; (C) Calcein; (D) No indicator; and (E) Visualisation of the products from (D) with ultraviolet light after ethidium bromide staining. RIME-LAMP was performed for this experiment. All indicators, and electrophoresis result showed 0.01 parasites/tube detection limits, which means that the new indicator, GelGreen, did not inhibit the LAMP reaction at all, nevertheless it detected amplified DNA directly.
(TIF)

S3 Fig Cross reactivity check of RIME-, 18S-rRNA-,and SRA-LAMP primers. The LAMP primer sets was tested for cross-reactivity with purified DNA (10 pg) from related species. Tbr: *Trypanosoma brucei rhodesiense* (UTH 2012); Tbg: *Trypanosoma brucei gambience* (IL2343); TcY: *Trypanosoma cruzi* (Y); TcT: *Trypanosoma cruzi* (Tulahuen strain); Leish: *Leishmania donovani* (MHOM/SU/62/2S-25M-C2); Pf: *Plasmodium falciparum* (3D7); Hu: Human blood DNA from a healthy individual.
(TIF)

S4 Fig Comparison of detection sensitivities of the SRA primer sets. New SRA primers detected 0.1 parasites per tube, while the old SRA primers were ten times less sensitive for Zambia strain.
(TIF)
