# Supplementary figures and images for: Direct Blood Dry LAMP: A Rapid, Stable, and Easy Diagnostic Tool for Human African Trypanosomiasis
Source: PLoS Negl Trop Dis. 2015 Mar 13;9(3):e0003578. doi: 10.1371/journal.pntd.0003578 (PMC4358998; doi:10.1371/journal.pntd.0003578)

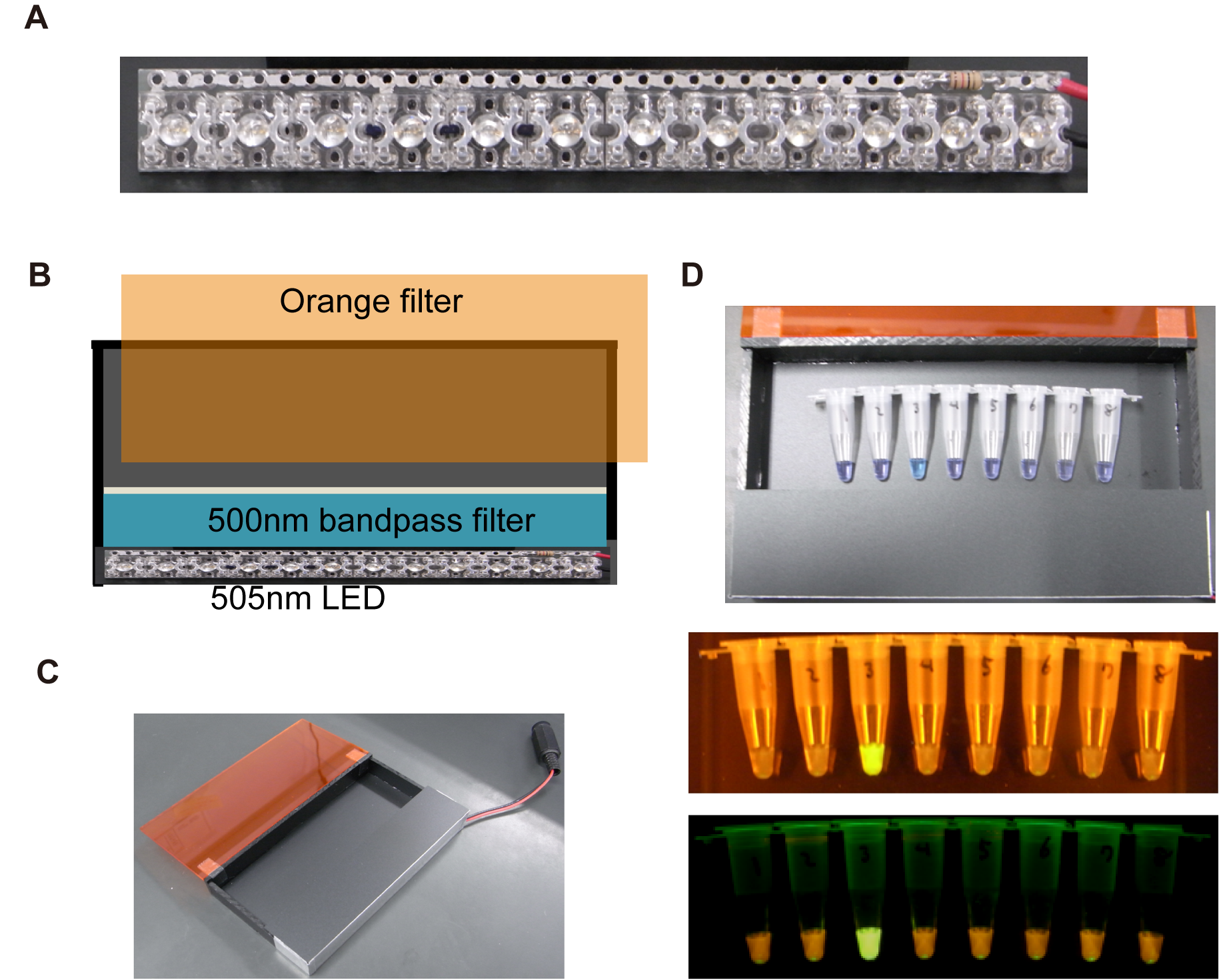

Supplement: S1 Fig — (B) Diagrammatic illustration of the portable RD. Emission light was coalesced into 500 nm wavelength by a gelatin film band path filter (FUJIFILM BPB-50). (C) Overall picture of LAMP RD. (D) Views of sample tubes in the RD. Naked eye (upper), emitted in the lighted (middle) or in the dark (lower) environment. The third tube from the left is reaction positive and the others are all negative. (TIF) [file pntd.0003578.s001.tif]

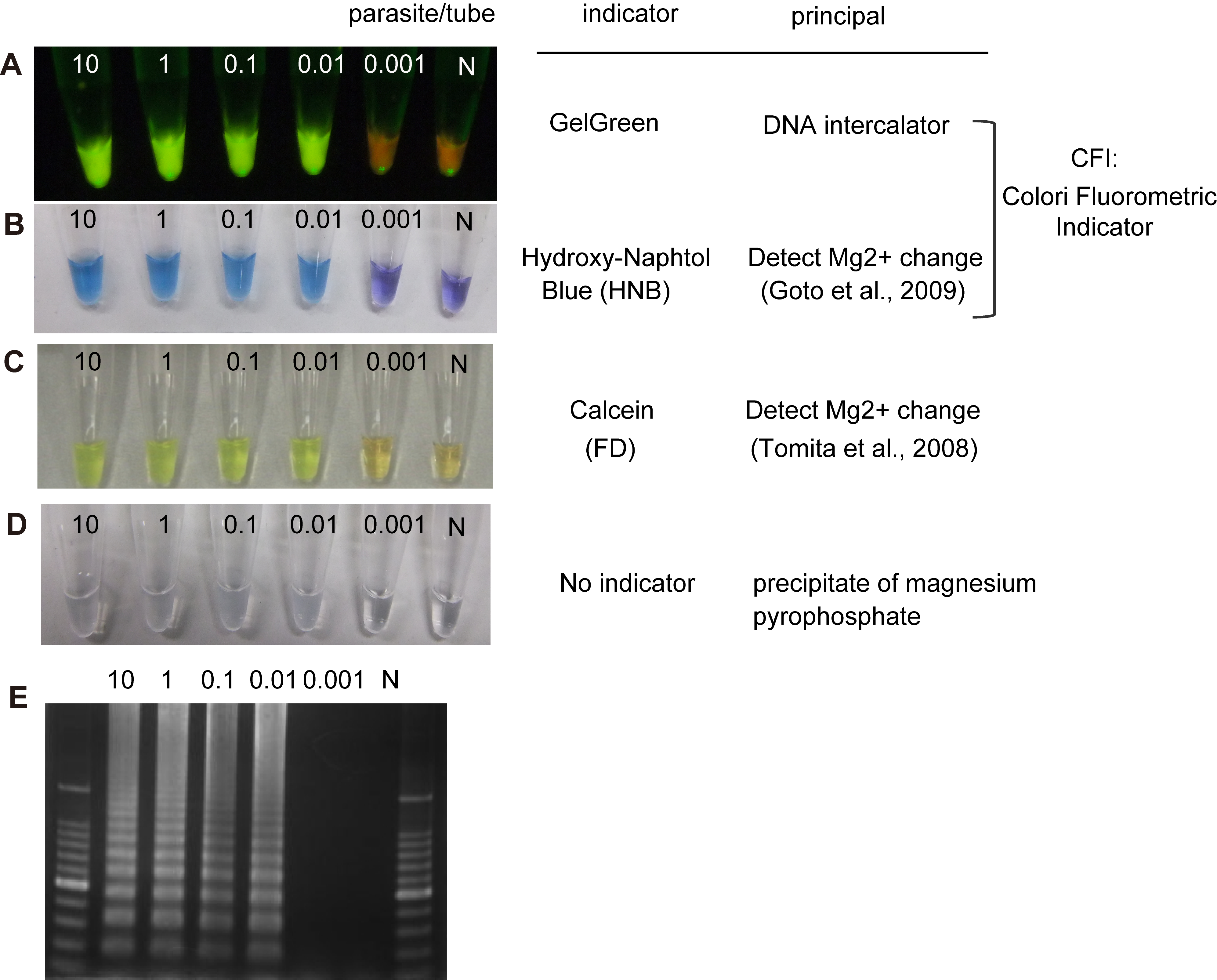

Supplement: S2 Fig — (A) GelGreen; (B) Hydroxy-naphtol blue; (C) Calcein; (D) No indicator; and (E) Visualisation of the products from (D) with ultraviolet light after ethidium bromide staining. RIME-LAMP was performed for this experiment. All indicators, and electrophoresis result showed 0.01 parasites/tube detection limits, which means that the new indicator, GelGreen, did not inhibit the LAMP reaction at all, nevertheless it detected amplified DNA directly. (TIF) [file pntd.0003578.s002.tif]

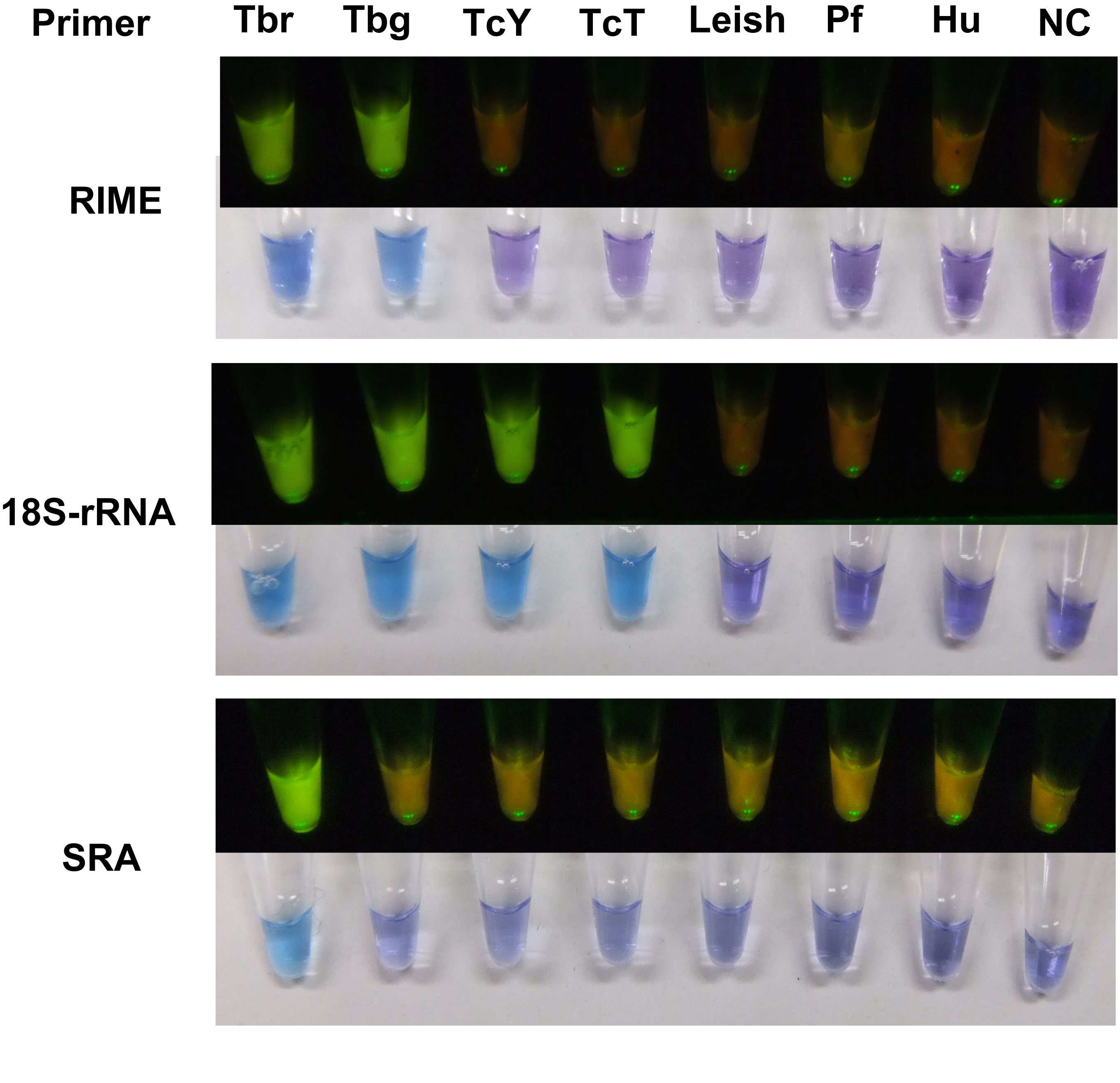

Supplement: S3 Fig — The LAMP primer sets was tested for cross-reactivity with purified DNA (10 pg) from related species. Tbr: Trypanosoma brucei rhodesiense (UTH 2012); Tbg: Trypanosoma brucei gambience (IL2343); TcY: Trypanosoma cruzi (Y); TcT: Trypanosoma cruzi (Tulahuen strain); Leish: Leishmania donovani (MHOM/SU/62/2S-25M-C2); Pf: Plasmodium falciparum (3D7); Hu: Human blood DNA from a healthy individual. (TIF) [file pntd.0003578.s003.tif]

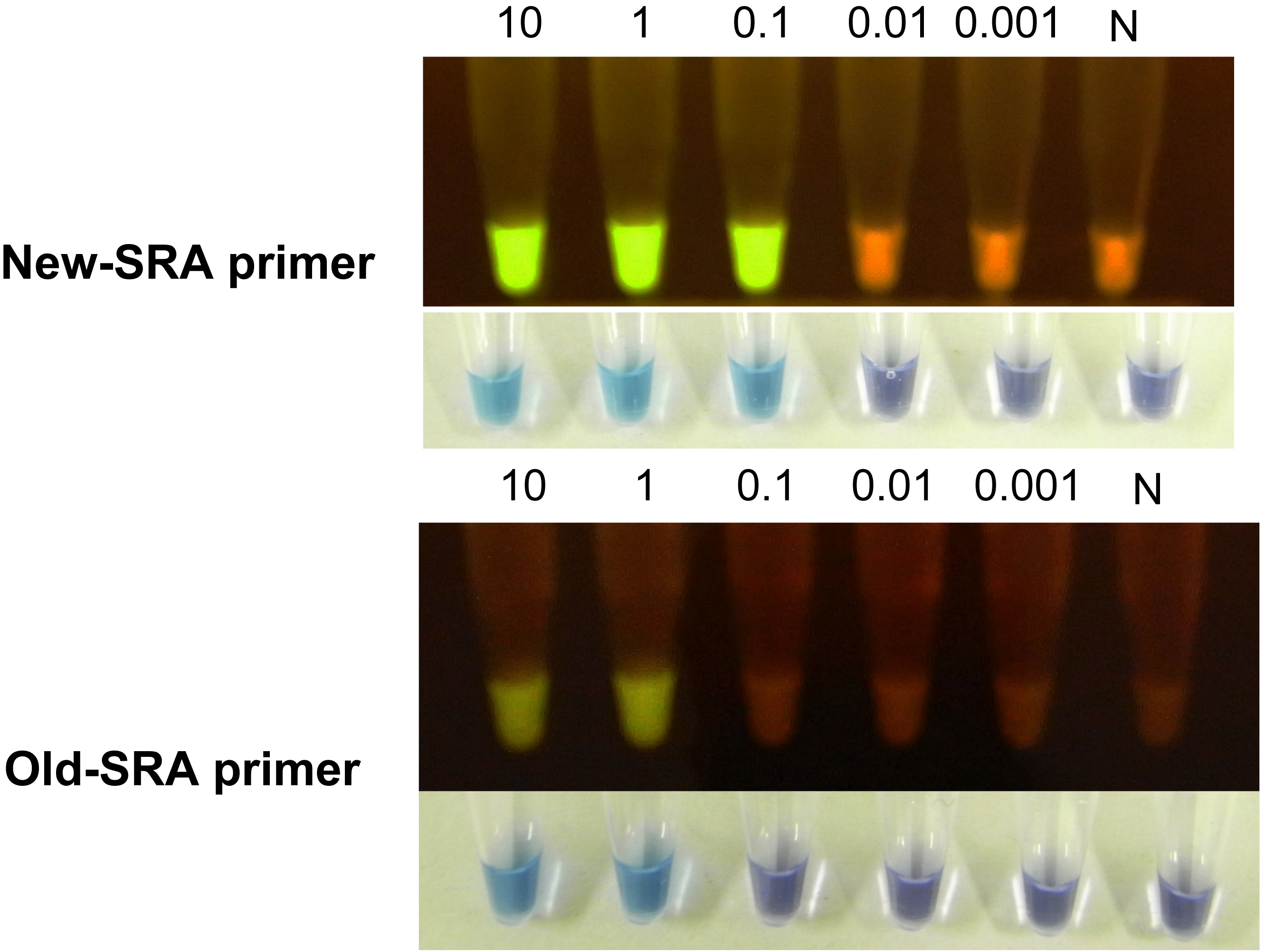

Supplement: S4 Fig — New SRA primers detected 0.1 parasites per tube, while the old SRA primers were ten times less sensitive for Zambia strain. (TIF) [file pntd.0003578.s004.tif]
